# Organization of Pediatric Echocardiography Laboratories: Impact of Sonographers on Clinical, Academic, and Financial Performance

**DOI:** 10.3389/fped.2022.891360

**Published:** 2022-05-30

**Authors:** Nick Arbic, Maelys Venet, Xavier Iriart, Andreea Dragulescu, Jean-Benoit Thambo, Mark K. Friedberg, Vitor Guerra, Conall Thomas Morgan, Luc Mertens, Olivier Villemain

**Affiliations:** ^1^Division of Cardiology, Department of Pediatrics, The Hospital for Sick Children, University of Toronto, Toronto, ON, Canada; ^2^Department of Pediatric and Adult Congenital Cardiology, Hôpital Cardiologique du Haut-Lévêque, CHU de Bordeaux, Bordeaux, France; ^3^IHU LIRYC Electrophysiology and Heart Modeling Institute, Fondation Bordeaux Université, Bordeaux, France

**Keywords:** pediatric echocardiography, laboratory management, sonographers, pediatric cardiology, congenital heart disease

## Abstract

Echocardiography has evolved the first-line imaging for diagnosis and management of pediatric and congenital heart disease all over the world. While it recognized as essential component of pediatric cardiac care delivery, organization of pediatric echocardiography services is very heterogeneous across the world, mainly related to significant differences in material and human resources in heterogeneous health care systems. In this paper, we focus on the role of pediatric sonographers, defined as expert technicians in pediatric echocardiography. While in some services sonographers are an essential part of the organizational structure, other laboratories operate only with physicians trained in echocardiography. The impact of sonographers on clinical, academic and financial performance will be discussed. Two organizational models (with and without sonographers) will be compared, and the advantages and disadvantages of each model will be evaluated. Different models of care provision are possible and decisions on organizational models need to be adjusted to the demands and available resources.

## Graphical Abstract

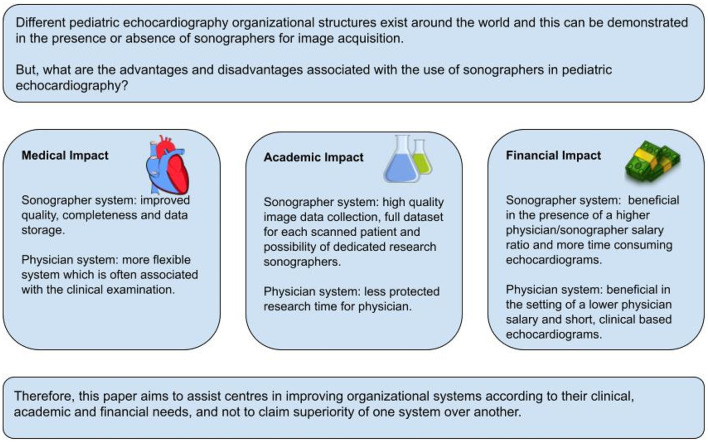

## Introduction

Since cardiac ultrasound was introduced in the 1950s (1), echocardiography has become the first-line imaging modality in cardiology, essential for the diagnosis of acquired and congenital heart disease (CHD). This success is based on echocardiography being a readily available bedside non-invasive real-time technique, that is relatively inexpensive compared to other imaging modalities such as cardiac MRI, CT, and cardiac catheterization. Since it was introduced in clinical practice, its further success was driven by technological innovation as well as by basic and clinical research validating its diagnostic utility. Pioneering work in the 1970s–1980s led to major breakthroughs in non-invasive diagnosis CHD, allowing accurate neonatal and even prenatal diagnosis of most congenital heart defects, earlier diagnosis and improvement in patient management (2).

As the use of pediatric echocardiography expanded, different organizational systems in pediatric cardiology have evolved around the world. Pediatric echocardiography training became an essential component of pediatric cardiology training programs and all pediatric cardiologists should be competent in performing and interpreting echocardiographic studies in children. In some country’s technician training programs specific for pediatric and adult echocardiography developed and the cardiac sonographer profession was introduced to assist with image acquisition and interpretation. All pediatric cardiology services provide echocardiographic imaging, but the organizational structures can be different between countries or even within countries. Specialized pediatric echocardiography laboratories developed in the 1980s–1990s and pediatric echocardiography became a recognized subspecialty in many countries different organizational models for pediatric echocardiography developed in different healthcare systems. So, for instance, in Canada, image acquisition is mainly performed by specialized pediatric sonographers, with image interpretation mainly done by pediatric cardiologists. In many other countries, pediatric cardiologists do the image acquisition, interpretation and reporting. Each organizational system has its advantages and disadvantages with impact on patient care, training of young physicians, scientific productivity, and financial cost.

The objective of this review is to compare the clinical, academic and financial impact of both organizational systems (with or without sonographers). We believe this could help individual centers to choose the model according to their clinical needs, academic objectives, and structural and financial resources.

## History of the Sonographer’s Role and Unique Considerations for Pediatric Sonographers

The sonographer profession began appearing in the United States in the early 1960’s around the time as the inception of clinical echocardiography. In 1970, the American Society of Ultrasound Technical Specialists (ASUTS) was founded by the American Institute of Ultrasound in Medicine (AIUM) (3). With the rapid growth of the ultrasound field, the need for formal guidelines and training requirements became crucial. In 1975, the sonographer profession accreditation process was formalized by the creation of the American Registry of Diagnostic Medical Sonographers (ARDMS). Currently ARDMS is still responsible for credentialing sonographers in the United States and its accreditation process is also recognized in most parts of Canada. Official training guidelines for cardiac sonographer education are regularly updated (4). Currently, The Hospital for Sick Children (SickKids), which is the largest pediatric heart center in Canada, employs 12 registered clinical pediatric cardiac sonographers and 4 research sonographers.

**FIGURE 1:**
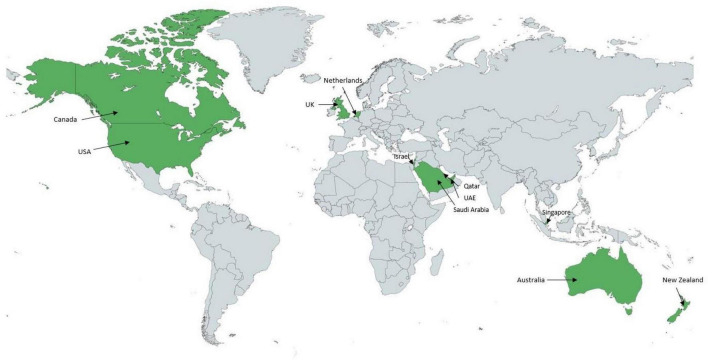
World map representing the countries (in green) operating with a predominant sonographer model in the acquisition of pediatric echocardiograms. Applies to 11 countries to date.

While in many countries around the world sonographers are working in adult cardiology services, fewer institutions employ pediatric cardiac sonographers. To our knowledge, 11 countries use a sonographer-based structure in pediatric cardiology (Figure 1). While in the North American health care system, pediatric cardiac sonographers have been well integrated for the last forty years, pediatric cardiologists perform pediatric echocardiograms in the majority of countries around the world.

The complexity of CHD requires extensive specialized training to become a proficient pediatric cardiac sonographer (5). For example, in Canada, current training to become a pediatric cardiac sonographer involves several steps: completion of a recognized diagnostic cardiac sonography college graduate, application for credentialing and practical training. Performing echocardiography in pediatric cardiology requires a high level of technical and interpretation skills provided by specialized training and continuous education to maintain diagnostic accuracy (5).

## Description of the Two Current Systems: Physician vs. Sonographer Models

Our description is based on two sample hospitals and the authors personal experiences moving between centers. This includes the University Hospital of Bordeaux, Aquitaine, France (*physician model*) and the Hospital for Sick Children (SickKids), Toronto, Ontario, Canada (*sonographer model*). Based on the databases of the European Congenital Heart Surgeons Association (ECHSA) and the Society of Thoracic Surgeons (STS), postoperative mortality for CHD is broadly comparable between North America and Europe (6–8). These figures do not represent all the care services provided but we assume that France and Canadian organizations are close in terms of clinical outcomes in pediatric cardiology.

### Sonographer-Based Organizations (*Sonographer Model*)

**FIGURE 2:**
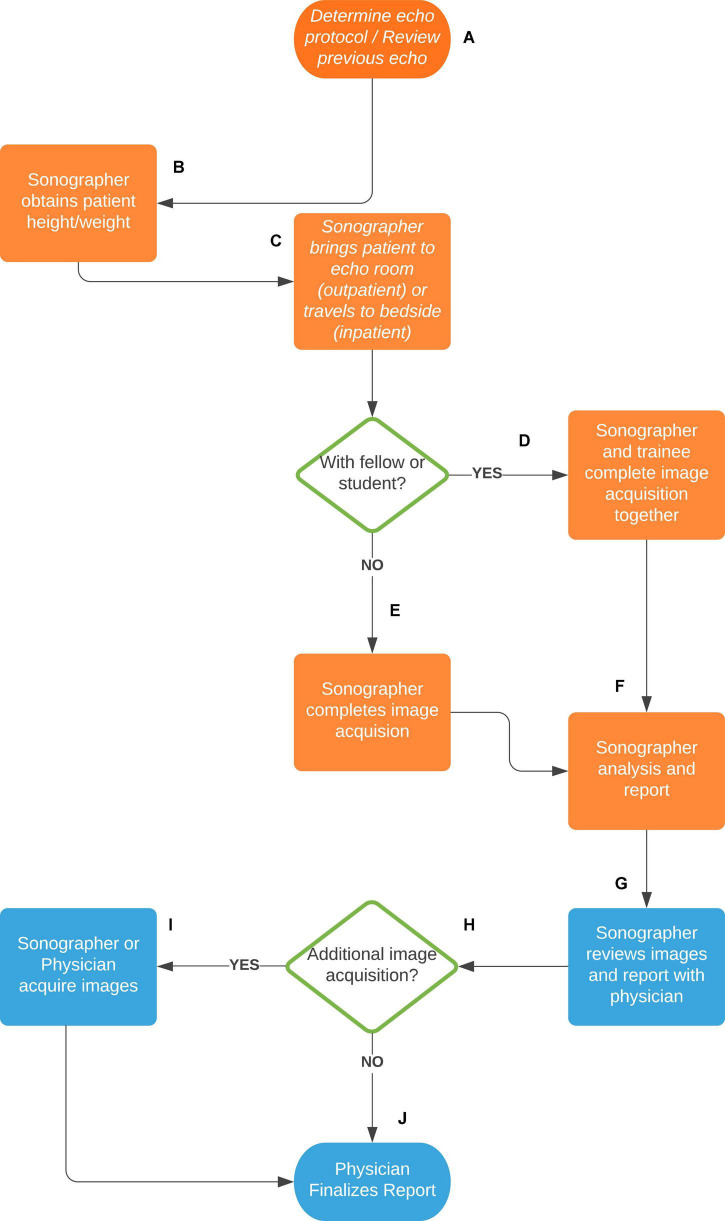
Sonographer-based scanning system at the Hospital for Sick Children of Toronto, Canada. **(A)** The sonographer reviews the patient echocardiography order and previous echo images to determine the appropriate scanning protocol. **(B)** The sonographer obtains the patient height and weight for growth and z-score calculations. **(C)** For an outpatient or mobile inpatient, the sonographer brings the patient into the echo scanning room. For a non-mobile inpatient, the sonographer brings the machine to the patient bedside. **(D)** If the sonographer is with a trainee (fellow or student), both complete the image acquisition. **(E)** Without a trainee the sonographer completes the image acquisition alone. **(F)** After image acquisition, the sonographer reviews them and completes a preliminary echocardiography report, including necessary measurements, findings, conclusions, and diagnostic coding. **(G)** The sonographer then reviews the images and report with a pediatric cardiologist. **(H)** The cardiologist determines whether any additional images need to be acquired. **(I)** If yes, the sonographer or cardiologist will acquire additional images. **(J)** If no, or following the additional images, the cardiologist finalizes the echocardiography report.

In SickKids, echocardiograms are performed by a specialized Pediatric Echocardiography Laboratory supervised by pediatric cardiologists with subspecialty training in pediatric echocardiography. The unit has 12 clinical sonographers and 4 research sonographers. The sonographers are responsible for image acquisition of outpatient and inpatient transthoracic echocardiograms (TTE). Image acquisition is guided by detailed imaging protocol that is disease-specific and differ for different stages. Each sonographer follows the same protocol during the image acquisition to guarantee that all essential cardiac structures are imaged with the same standardized views and the same measurements are obtained. After acquisition, the images are reviewed and discussed with the attending staff and sometimes additional images can be acquired either by the sonographer or the physician. The sonographer drafts a preliminary report that is amended and signed off by the physician who takes the final diagnostic responsibility. Completion of a pediatric echocardiogram in a sonographer-based scanning system involves sonographer and cardiologist interaction and collaboration (Figure 2).

Over the past 5 years at SickKids, an average of 13,386 TTE are performed each year. With around 12 full time sonographers, the annual average number of pediatric TTE per sonographer is 1,134. These numbers are comparable with other large North American surgical centers (9). The sonographer input allows one physician to report more TTE per day compared to a system where the physician performs the study. The process, however, is lengthier with each study raging between 15 min for a limited assessment to more than 1 h for more complex pathology. Most follow-up TTE are scheduled in 60-min slots.

### Physician-Based Organizations (*Physician Model*)

**FIGURE 3:**
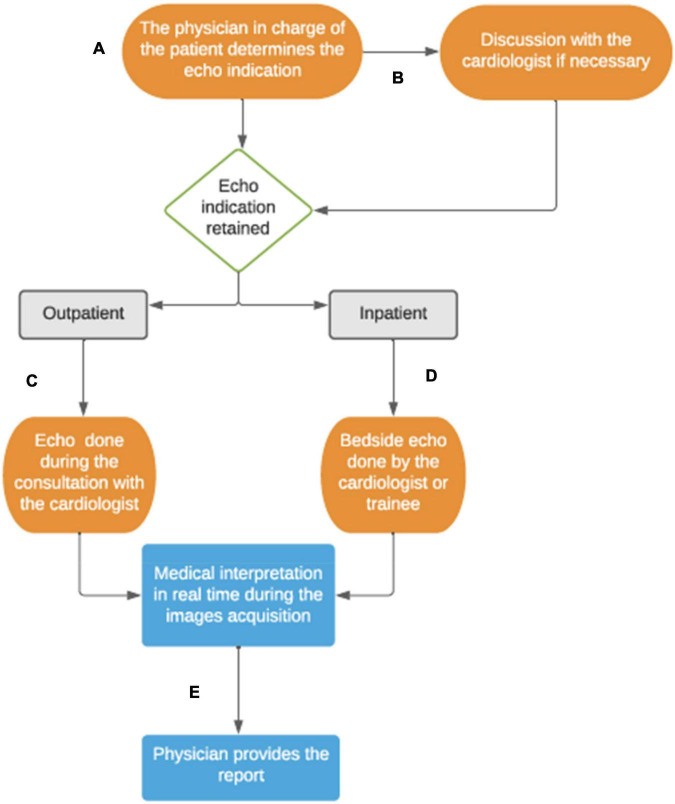
Physician-based echocardiography system at the University Hospital of Bordeaux, France. **(A)** The physician assesses the echocardiogram indication for his patients. **(B)** If the physician is not a cardiologist, he usually discusses with the heart team to validate the indication. **(C)** For the outpatients followed up in cardiology clinic, the echocardiogram is done by the cardiologist during the consultation. **(D)** About inpatients, the inward cardiologist staff and trainees manage the TTE indication and scanning during the hospitalization. **(E)** The interpretation of the images is done in real time and the report is noted with the patient’s daily notes, or in the consultation letter for outpatients.

We use the Bordeaux center as an example of a pediatric cardiology service operating without sonographer. In this model, all pediatric cardiologists and trainees perform echocardiograms. Whether the physician was primarily trained in pediatrics or whether adult cardiology, pediatric cardiology training includes an extensive echocardiography course and accreditation through a national diploma. This inter-university degree of echocardiography is obtained after regular theoretical course over 2 years, passing the related exam and achieving more than 120 echocardiograms per year in first operator, including transesophageal echocardiograms (10). In the *physician model*, the TTE is more considered as an extension of the clinical examination and every pediatric cardiologist acquires images, interprets and reports the findings. Therefore, the TTE is usually not performed in a dedicated room such as an echo room. For outpatients typically, the TTE is performed during the clinic visit by the patient’s cardiologist, in the clinic examination room. For inpatients, physicians and trainees staffing the wards perform bedside echocardiograms when needed, usually as part of the inpatient round, without approval from an extraneous Echo Lab (Figure 3). Overall, this system decreases the time of use of the ultrasound machine but it also allows more flexibility for the physician to decide on the use of the ultrasound system and its availability. In this setting, the physicians also take responsibility for interpreting and documenting the results.

The University Hospital of Bordeaux is a national referent center in CHD in France with a similar type of activity to that of SickKids, with the same overall percentage of patients referred from peripheral centers and 280 pediatric bypass surgeries done annually (vs. 450/year in SickKids). Seven pediatric cardiologists are involved in performing the pediatric echocardiography, in addition to their other clinical duties (e.g., outpatient clinic, inpatient activity, consultations, catheterization). From 8,500 to 9,000 pediatric TTE are performed each year. The echocardiogram acquisition is protocolized, images are saved, and independent electronic reports provided for all outpatients. The average time spent per echo is much lower than in centers with sonographers, about 20 min per examination for outpatients and about 5–10 min for daily follow-up of hospitalized patients. However, the anatomical studies for the initial diagnosis of congenital heart disease are just as complete as described in the sonographer model, based on segmental analysis and exhaustive study of each anatomical structure.

### Hybrid Organizations

Many centers using a physician-based model transition into a hybrid model by recruiting sonographers to handle part of the scanning workload (11, 12). They can have different training backgrounds including nurses trained by physicians. Often the training is not standardized, and the performance can be variable, as well as its effective role in scanning and reporting. To our knowledge, hybrid models are not a widely used organization. Within these centers there are often only one or two technicians, covering a small part of the daily studies. However, health care institutions traditionally belonging to the physician model seem to be interested in developing an hybrid model (13), which could potentially be financially advantageous. Nevertheless, there is still a considerable lack of structural training and recognition for the pediatric cardiology sonographers in countries historically based on a physician model.

## Sonographer’s Impacts

### Medical Impact

The primary contribution of sonographers in the organization of an Echocardiography Laboratory is their impact on the medical practice.

First, the ability to perform a large quantity of pediatric echocardiograms, thanks to an optimal sonographer/patient ratio, helps to reduce wait times. Indeed, this is particularly marked for outpatients in comparison with systems where echocardiogram is performed during the medical consultation. In the SickKids setting, the non-urgent echo requests have mean wait times of about 1 week (internal data). This is made possible because capacity is built into the schedules to be able to absorb additional requests. For inpatient emergencies where fast access to echocardiographic imaging can be important, the physician-based system is usually more reactive. Typically, an ultrasound scanner is immediately available on the cardiac wards and the physician can perform it without any delays. The introduction of point-of-care ultrasound (POCUS) in intensive care units and emergency departments, indicates the need for immediate access to ultrasound technology and could partially address the need to have imaging information available for immediate decision making (14). Having access to expert echocardiographic skills can, however, be beneficial, especially for patients with complex heart disease (15). In addition to emergency situations, we observed between our two centers that the mean interval between the day of return home after bypass surgery and the first follow-up echo was similar (7.8 ± 3.4 days and 6.4 ± 2.8 days for SickKids and Bordeaux, respectively). This allows us to consider that the type of organization does not seem to have a direct impact on the rules of management of patients in stable situations.

Secondly, one of the key parameters to support the sonographer model in pediatric cardiology is the quality of echocardiograms. Echocardiography is a very operator-dependent skill and the ability to perform detailed pediatric echocardiograms requires proficient technical skills. Human performance studies indicated that in order to develop “expert” level performance for certain skills, can take years of dedicated practice (16) and up to 10,000 h of focused practice (17). The time needed to develop expert level pediatric echocardiographic skills likely highly varies between operators and has not been well studied. The focus of sonographers on image acquisition, certainly allows them to reach this technical “expert” level performance, especially in environments where teaching and continuous performance improvement is integrated in the work environment. Quality improvement (QI) programs are embedded in the organization of pediatric echocardiography laboratories and this includes standardization of practice through introduction scanning protocols and QI processes aimed at reducing inter-operator differences. Despite being protocol-based sonographers have the flexibility to adjust scanning protocols according to clinical needs and the review process is aimed at guaranteeing that all relevant information needed for clinical management is obtained and reported. As an example, the rate of echo repetition for lack of image acquisition has been estimated at 3% in SickKids (sonographer model) and at 11% in Bordeaux (physician model) [unpublished datas]. The systematized and highly protocolized approach of the sonographer model seems to allow a reduction of echo repetition.

As part of the QI-processes of any diagnostic imaging environment, consistent storage and reporting of all relevant images is integrated into the sonographer practice. This is less standardized in most physician-based environments where imaging information used for clinical decision-making may not necessarily be stored and easily accessed. This allows retrospective review and assess changes over time by comparing serial studies. Each echocardiogram can be *a posteriori* interpreted and discussed, as a cross-sectional imaging examination. This is particularly important in a specialty where most medical and surgical decisions are based on echocardiographic findings (18–21).

Building strong sonographer-physician interactions and creating strong interprofessional collaboration improves patient care. Increasing evidence links the quality of teamwork with quality and safety in healthcare delivery (22). The technical and interpretation expertise is shared on a continual basis within the team allowing each member to feel supported by their colleagues. From a practical perspective, there are always at least two sets of “expert eyes” on every echocardiogram completed. This can help to avoid mistakes or oversights (23). As with Reason’s Swiss cheese model, adding a participant in the chain adds a barrier against error (24). The counterpart of this process could be the incidental discovery of more anatomical abnormalities whose clinical significance is uncertain and management difficult in terms of therapeutic decisions or follow-up modalities (25). Overall, the team-work component of a sonographer-based model works to promote diagnostic accuracy and therefore, improves the medical management of pediatric cardiology patients.

### Academic Impact

Academic pediatric cardiology centers play an essential role in education and innovation through research. Sonographers have both a direct and indirect impact on research. In centers such as the Hospital for Sick Children, where sonographers perform the majority of pediatric echocardiograms, there is protected research time for physicians. The scanning portion of a pediatric echocardiogram is the most time-consuming task and removing this role from the physician allows for more focus elsewhere. Knowing that physician research productivity increases with dedicated research time (26), the sonographer’s activity has an indirect impact on the team’s or unit’s research productivity.

Furthermore, protocol-based scanning has a direct impact on clinical research data. The ability to acquire the same data set for each protocol improves the quantity and quality of data for retrospective research use. For prospective studies, this further reduces variability and improves consistency of the collected images.

The research sonographer team at the Hospital for Sick Children consists of four sonographers, a dedicated research space and specific research ultrasound equipment. A dedicated team of research sonographers provides many benefits to research productivity and quality. Firstly, most research sonographers are experienced clinical sonographers, who bring with them a high level of technical proficiency in image acquisition. Secondly, a dedicated team of research sonographers can analyze a large quantity of data. This allows the unit to be involved in a large quantity of projects as they have a dedicated team assigned to provide high quality analysis. Lastly, they are involved in the organization of research projects, including patient scheduling and data storage. Providing an organizational component to ongoing projects allows for better research efficiency and saving medical time (27).

### Financial Impact

**FIGURE 4:**
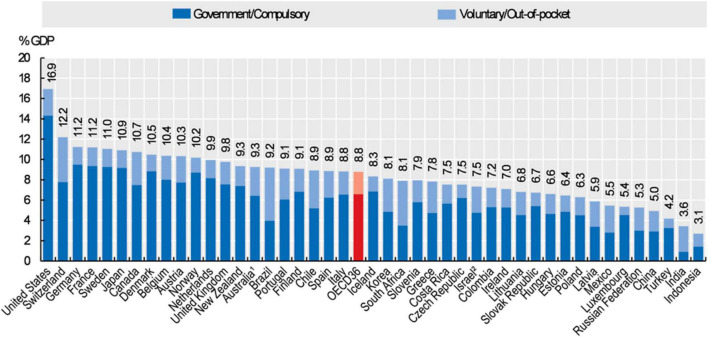
Health expenditures as a proportion of the Gross Domestic Product (GDP) in 2019. Source: 2019 OECD Health Statistics; WHO Global Health Expenditure Database.

The financial impact of different models and study of cost-effectiveness are essential in all healthcare environments as resources for healthcare differ widely between countries and even within different countries dependent on the region or even hospital. The healthcare systems in France and Canada, used in this example, share a lot of similarities with both having a system of universal health coverage largely financed by taxation (28). In both organizations, residents have reasonable access to medically necessary care provided in hospitals or by physicians, which explicitly includes diagnostic, treatment and preventive services. As far as pediatric cardiology care is concerned, the costs are fully covered by the French and Canadian healthcare systems. Both countries devoted a similar amount of their gross domestic product to healthcare spending, with Canada spending 10.7% and France spending 11.2% in 2019 (Figure 4) (29). With comparable universal healthcare delivery and spending, can an argument be made for a direct financial impact of the sonographer?

Within an individual hospital, the cardiac sonographer salary can have direct financial impact. At SickKids, the average ratio of cardiologist salary to sonographer salary is approximately 4/1.

The full billing cost of a pediatric echocardiogram in Canada is 212.80 Canadian dollars (CAD), with a technical component (116.60 CAD) or image acquisition billing cost and a professional component (96.20 CAD) or image review and reporting. Comparatively, the cost of a cardiac echocardiography Doppler in France is fixed at 96,49 euros (≈ 140 CAD) according to the 68th version of the “*Classification Commune des Actes Médicaux*” in charge of the procedure coding in France. In a sonographer model, the technical component corresponds to the sonographer role and the professional component to the physician role. Image acquisition is the most time-consuming portion of a complete pediatric echocardiogram. Thus, it would be financially beneficial to have the lower salaried sonographer complete the more time-consuming task. This allows a single physician to review and report 25–30 echocardiograms per day (internal data from SickKids), which efficiently makes use of the professional component. Given the duration of a pediatric echocardiogram and the current cost of billing in Canada, the sonographer is used as a less expensive workforce to make the most of medical time and save on tasks that could be performed by non-medical technicians. This is particularly attractive in a Canadian system where medical time is preserved and where doctors’ salaries are about three times the salary of French public hospital’s physicians (30). Thus, the profitability of the model with or without sonographer is necessarily dictated by the difference in salaries of the different actors and the time required per echocardiogram. However, it is rather beyond the scope of this paper to determine which model is more cost effective. It depends a lot on the structural organization of each country, in terms of medical salaries and effective working time. The more protected time a physician has and the higher the salaries are, the more the financial contribution of the sonographers is cost effective.

## Discussion

**FIGURE 5:**
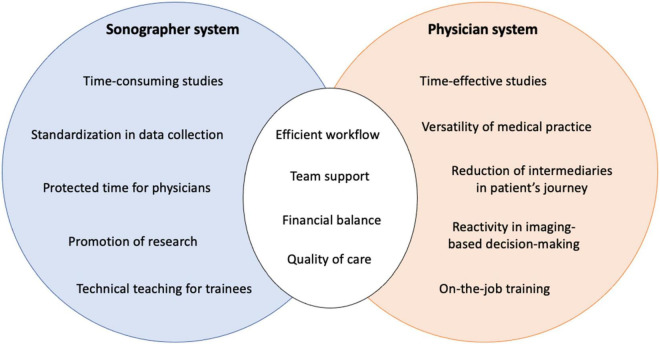
Strengths, weaknesses and common objectives for the sonographer and the physician systems.

The interest of comparing these models is not limited to the financial question. As described above, from a purely qualitative point of view, the echocardiogram acquisitions could appear more complete and protocolized through the sonographer model and the sonographer contribution to quality of care and research has been detailed. However, other points have to be discussed to better understand all the dimensions affected by these organizations (Figure 5).

### The Challenge of the Sonographer Performance

For a system like the one developed at SickKids, the key parameter is the high-quality training and skills of the sonographers. Indeed, the added value can only be imagined if the sonographer has a high degree of autonomy and understanding of pediatric and congenital cardiology. Physicians must be able to analyze the sonographers’ images for the time saving to be real. Our comments are based on the SickKids experience, where the training and competence of the sonographers is a reference. However, sonographer training remains heterogenous according to individual centers and this must be considered in the appreciation of the sonographer model.

### Conditions of Work for Physicians

The question of burn out among physicians is a well-known problem affecting the workforce worldwide. As Scott. W. Yattes wrote in a recent review paper: “Burnout is a system problem, not an individual disease and must be addressed with systematic solutions” (31). The literature systematically reports the same causes leading to this phenomenon: in the first rank, the long working hours, the work overload and the wasting-time in non-medical tasks (32–36). The extent and consequences of medical burn out are terrible, affecting approximately one-half of physicians in public hospitals and can result in medical errors, lower quality of care, higher costs, and poor team dynamics (37). Recruiting sonographers could be more easily acceptable to the administration than recruiting physicians in the context of the lack of public health means in most European countries (38, 39). Good distribution of tasks and the collaborative work between physician and technicians should relieve the physician workload and must be considered to improve healthcare wellness, such as administrative support in another topic (35, 36, 40).

### System Efficiency

In the sonographer model, the time between the TTE request and the receipt of the signed report by the medical team in charge of the patient is longer than when the physician directly scans his own patients. This can lead to delay in decision-making where the TTE findings are crucial (41). Thus, the reactivity of this organization necessarily depends on good communication among its members and efficient prioritization of the most urgent studies.

Finally, our opinion is that the system efficiency depends largely on the Echo Lab management rather than on the presence or absence of a sonographer. Efficient team management promotes a safe and fulfilling work environment and results in less turnover and better global skills and productivity.

### Impact on the Patient

The duration of examination in children can be a significant barrier to compliance, comfort and indirectly image quality. In some centers such as SickKids, this leads to provide sedated echocardiography in patients from 3 weeks to 3 years of age to be able to complete the whole protocol. This means spending at least half a day in the hospital, and even when the echocardiogram is not under sedation, the echo appointment is separated from the medical appointment, which lengthens the patient’s journey. An additional potential consequence of the sonographer system is the multitude of people involved in the care pathway. The experience for the family is necessarily different when the referent physician, following the child in the long term, performs the echocardiograms during the medical consultation and then gives the result immediately after, in the same unit of time and place.

## The Future of Echocardiography and Impact of Novel Technology on Workforce

The sonographer profession supports the importance of ultrasound image acquisition, not its final interpretation. The shortcoming of past and present ultrasound image acquisition is that it remains mainly operator dependent and therefore, requires a trained and skilled professional. However, with the continued rapid development of technology, including 3-dimensional echocardiography, artificial intelligence (AI) and robotization, it is reasonable to envision a future system where ultrasound image acquisition could be fully automated.

3D echocardiography (3DE) has been slow to integrate as a routine diagnostic tool. Historically, the central limitation of 3DE has been its inferior image resolution, both temporal and spatial, when compared with 2-dimensional (2D) imaging. However, improvements in probe technology now allow for higher spatial resolution and volume rates needed for cardiac imaging (42). In addition, today’s single matrix array transducers have the ability to generate excellent 2D and 3D image quality, eliminating the need for separate transducers and allowing 3DE to be more easily integrated. There are several technological advancements in 3DE that could change the sonographer’s role in pediatric echocardiography. First, fusion of 3DE with other imaging modalities such as computed tomography (43) and fluoroscopy (44) can provide a better 3-dimensional understanding of anatomical relationships. Second, the ability to utilize 3DE to provide reliable 3D printing of heart structures such as atrioventricular and semilunar valves (45) now allows for more intuitive cardiac information. Lastly, 3DE can now be displayed in virtual reality or holograph, providing structural information that is not be appreciated in standard 2-dimensional echocardiography (46). The ongoing developments in technology continue to grow the capacity of 3DE in pediatric cardiology. It is conceivable that with the continued growth of 3DE, a sonographer’s role in healthcare could evolve to be primarily a post-processing job.

The field of robotization has also begun its integration into echocardiography with the development of tele-operated robots for remote studies. One of the first robot operated echocardiography systems was developed for use in space in order to monitor the effects of space travel on the cardiovascular system. The Tele-echography for the European Space Agency (TESSA) was designed to allow trained experts on the ground to operate a remote echocardiography system and obtain real-time images. The TESSA system consists of an ultrasound machine, an ultrasound probe attached to a robotic arm and a video conferencing system (47). These robotic echocardiography systems allow a trained professional to control the probe remotely, via a joystick. Presently, technology such as the TESSA is used primarily for space travel, however it could also benefit other remote locations such as rural areas. With continued evolution of robotization in echocardiography, the distant future could see sonographers may be replaced.

Lastly, artificial intelligence (AI) in echocardiography is an ever-expanding field that has the potential to reduce the need for trained sonographers. Artificial intelligence is a computer-based system that can observe its environment and take actions to successfully achieve its goals. Currently there are subsections of AI -machine learning (ML) and deep learning (DL)- that are used to enhance acquisition, analysis and reporting in echocardiography. In its simplest form, ML is centered around computers being able to think and act with minimal human involvement; however, DL is about computers learning to think utilizing structures modeled on the human brain. For the purposes of this paper, we will focus only on the use of AI in image acquisition as this is primarily the sonographer’s role. First, new AI software has been studied and developed to assist the user in acquiring good quality echocardiographic images (48). Recently the Food and Drug Administration (FDA) approved the first AI echocardiography software designed to guide the user to adjust the probe for the acquisition of diagnostic quality images (49). The software was developed using machine learning in order to recognize acceptable image quality and automatically save the best video clip from each particular view. In addition, current DL software has been shown to perform well in echocardiographic view classification (50). These two types of AI software allow users with limited to no prior echocardiography experience to obtain diagnostic cardiac views.

## Conclusion

Determining the superiority of one model over the other is not the goal of this study. This work aims to highlight the advantages and disadvantages of both. What distinguishes these two models does not only imply a different Echo Lab organization but involves a different history, culture and concept of care. Transposition from one system to another is neither desirable nor possible but highlighting areas of progress can help actors analyze their system differently, in order to constantly improve the quality of care and working environment. We believe this work will help teams improve their operations by leveraging the advantages and strengths of each system, possibly evolving toward a balanced hybrid system adapted to each situation.

## Author Contributions

All authors listed have made a substantial, direct, and intellectual contribution to the work, and approved it for publication.

## Conflict of Interest

The authors declare that the research was conducted in the absence of any commercial or financial relationships that could be construed as a potential conflict of interest.

## Publisher’s Note

All claims expressed in this article are solely those of the authors and do not necessarily represent those of their affiliated organizations, or those of the publisher, the editors and the reviewers. Any product that may be evaluated in this article, or claim that may be made by its manufacturer, is not guaranteed or endorsed by the publisher.
